# Sweet corn association panel and genome-wide association analysis reveal loci for chilling-tolerant germination

**DOI:** 10.1038/s41598-024-61797-7

**Published:** 2024-05-11

**Authors:** Zhenxing Wu, Tingzhen Wang, Jianjian Chen, Yun Zhang, Guihua Lv

**Affiliations:** 1 Institute of Maize and Featured Upland Crops, Zhejiang Academy of Agricultural Sciences, Dongyang, 322100 China; 2 Horticultural Research Institute, Jilin City Academy of Agricultural Sciences, Jilin, 132000 China

**Keywords:** Sweet corn, Genome-wide association study (GWAS), Chilling-tolerant germination, SNP, Genetics, Plant sciences

## Abstract

Sweet corn is highly susceptible to the deleterious effects of low temperatures during the initial stages of growth and development. Employing a 56K chip, high-throughput single-nucleotide polymorphism (SNP) sequencing was conducted on 100 sweet corn inbred lines. Subsequently, six germination indicators*—*germination rate, germination index, germination time, relative germination rate, relative germination index, and relative germination time—were utilized for genome-wide association analysis. Candidate genes were identified via comparative analysis of homologous genes in Arabidopsis and rice, and their functions were validated using quantitative real-time polymerase chain reaction (qRT-PCR). The results revealed 35,430 high-quality SNPs, 16 of which were significantly correlated. Within 50 kb upstream and downstream of the identified SNPs, 46 associated genes were identified, of which six were confirmed as candidate genes. Their expression patterns indicated that *Zm11ΒHSDL5* and *Zm2OGO* likely play negative and positive regulatory roles, respectively, in the low-temperature germination of sweet corn. Thus, we determined that these two genes are responsible for regulating the low-temperature germination of sweet corn. This study contributes valuable theoretical support for improving sweet corn breeding and may aid in the creation of specific germplasm resources geared toward enhancing low-temperature tolerance in sweet corn.

## Introduction

Sweet corn, valued by consumers for its juicy texture, sweetness, and high nutritional value^1^, faces an escalating threat to production because of the heightened frequency of extreme weather events resulting from global climate change in recent years. One of the most significant challenges encountered during early spring cultivation is low-temperature stress^2,3^. Being a typical tropical and subtropical crop that thrives in warm temperatures, sweet corn exhibits reduced germination rates and low-quality crop yield when exposed to low temperatures^4,5^.

Low-temperature stress is categorized into cold stress (< 15 ℃) and freezing stress (< 0 ℃). Corn, representative of C4 plants, is particularly susceptible to cold stress, and germination rates are significantly affected at temperatures of 15 ℃ or lower^5,6^. Seed germination is a multifaceted process involving the reactivation of essential cellular events, including various metabolic reactions and signal transduction pathways^7–9^. Exposure to low temperatures induces physiological and biochemical changes in corn seeds, encompassing alterations in salicylic acid^10^, glycine betaine^11^, antioxidant enzymes^12^, Ca2^+^ influx^13^, and sugar efflux^14^. Moreover, low temperatures can detrimentally impact photosynthetic mechanisms^15^ and disrupt normal plant metabolism^16^. Although seed pretreatment employing chemicals such as ascorbic acid, salicylic acid^17^, melatonin^18^, and chitosan^19^ offers some relief from low-temperature damage to maize seed germination, it concurrently escalates seed costs. Therefore, genetic enhancements are being considered as a potentially more effective strategy to enhance seed vitality without increasing the cost.

Cold-tolerant germination characteristics among maize genotypes were initially documented in 1949^20^. In subsequent investigations, quantitative trait loci (QTL) related to cold related traits in maize have been studied. Mapping populations, based on F_2:3_ separated offspring of parents with differential phenotypes or recombinant inbred lines (RILs), were constructed for linkage analysis to identify major QTLs associated with chilling-tolerant germination^21–24^. Through these studies, some genes have been speculated to be related to seed low-temperature germination, such as genes encoding enzymes related to photosynthesis and ascorbic acid. However, limited by the parental mapping population, these findings are not very sensitive, with low resolution and a large QTL confidence interval. Advancements in high-throughput DNA sequencing technology have facilitated genome-wide association study (GWAS) analysis for screening complex quantitative trait genes in plants. Compared to linkage analysis, GWAS analysis offers a broader range of research subjects, higher genetic resolution, shorter processing times, lower costs, and more realistic associations^25–27^. In a study on 282 maize inbred lines, GWAS was conducted using high-throughput sequencing, simultaneously screening for a cold tolerance index during germination, and 18 potential candidate genes were identified^3^.

Despite the identification of some QTLs and genes related to chilling-tolerant germination in corn, similar studies on sweet corn are scant, leaving the underlying genetic mechanism unclear. This study addressed this gap by collecting 100 germplasm resources from sweet corn inbred lines, assessing germination indicators at room and low temperatures, and conducting high-throughput SNP sequencing, population structure, family evolution, and genotype–phenotype correlation analyses to identify closely related candidate genes. The outcomes may pave the way for cultivating new varieties of sweet corn with enhanced cold tolerance, foster a deeper understanding of the genetic principles governing cold tolerance in sweet corn, and offer a scientific reference for molecular marker-assisted selection in the low-temperature breeding of sweet corn.

## Materials and methods

### Plant materials and phenotypic assessment

The Institute of Maize and Featured Upland Crops of Zhejiang Academy of Agricultural Sciences, focuses on maize breeding as its main research direction, maintains germplasm resources from various regions worldwide. One hundred sweet corn inbred lines with different genetic backgrounds were meticulously selected as experimental materials, including tropical, temperate, subtropical, and American (inbred lines selected from American hybrids) materials. The inbred lines are listed in Supplementary Table S3. Seeds were sown in tropical Hainan Province, China during winter 2022 and in subtropical Zhejiang Province during spring 2023. After harvesting, seeds were dried to approximately 15% moisture content and stored below 2 ℃. Seeds of consistent size and plumpness were chosen, surface-sterilized in a 10% hydrogen peroxide solution for 30 min and rinsed with sterilized water. Thirty seeds per inbred line were then placed in a sterilized culture dish covered with a moist sponge and filter paper, repeated thrice, and incubated at 10 ℃ (chilling treatment) or 25 ℃ (control) under dark conditions for germination. Humidity was maintained by regular soaking with distilled water.

The number of germinated seeds were recorded following the national standard of China GB/T5520-2011 “Grain and Oil Inspection—Seed Germination Test.” The germination standard was met when the sprouts reached 1/2 of the grain length. For the control group (25 ℃), germination was monitored on days 1, 2, 3, 4, 5, 6, and 7. The germination rate (R), average germination time (T), and germination index (I) under room temperature were collected on day 7. For the chilling treatment group (10 ℃), the number of germinated seeds was observed from 0 to 21 days, and the germination rate (GR), average germination time (DT), and germination index (GI) under low temperature was calculated on day 21^28^.

Germination rate was calculated as: Germination rate = (total number of normally germinated seeds at the end of the germination period (within the specified dates)/number of tested seeds) × 100. The Germination index was calculated as: Germination index = ∑(Gt/Tt), where Tt is the number of days passed and Gt is the germination rate on the day t. Average germination time was calculated as: Average germination time = ∑(D × n)/∑n, where n is the number of newly germinated seeds on day D, and D is the number of days calculated from the germination test. In addition, the relative value of traits was calculated as follows: Relative value of traits = XL/XN, where XL represents the values measured under chilling stress (10 ℃), and XN represents the values measured under control (25 ℃). DPS v9.05 was used for phenotype analysis of variance, and the statistical model was the GLM (generalized linear model) model. R 3.12 was used for normality testing and data transformation of phenotype data and the R package ‘psych’ were utilized to determine the distribution and correlations among indicators related to chilling-tolerant germination of sweet corn seeds. We confirmed that we complied with international, national and/or institutional guidelines in our experiments on plants/plant parts.

### Genotyping data

Genomic DNA was extracted from young leaves of each of the 100 maize inbred lines using a modified cetyltrimethylammonium bromide (CTAB) method^29^. SNPs were detected using a 56K chip (Axiom Maize 56K SNP Array), which enabled high-throughput detection of 56,000 SNP sites. Genotyping analysis resulted in 38,613 high-quality SNPs (dish QC ≥ 0.82 and call rate ≥ 97)^30^. The software Plink 1.9 was used for data cleaning and filtering^31^. A total of 35,430 SNPs without minor allele frequency (MAF) < 0.05 and a missing data rate > 0.2 were filtered out for subsequent analysis.

The maximum-likelihood method in FastTree 2.1.9 was employed to construct a phylogenetic tree^32^, with iTQL (https://itol.embl.de/) used for coloration. PCA was conducted using GCTA (version 1.92.0), selecting the first two eigenvectors to generate a scatterplot of the PC scores for visualizing similarities among the accessions in two dimensions^33^. The relative kinship matrix (K) was generated using Tassel 2.1 software^34^, and R 3.12 was used for visualization to form a relative kinship heatmap. The linkage disequilibrium (LD) was determined using PopLDdecay software^35^.

### GWAS analysis

The chilling treatment germination rate (GR), chilling treatment average germination time (DT), chilling treatment germination index (GI), relative germination time (RDT), relative germination rate (RGR), and relative germination index (RGI) were used for GWAS analysis.

Phenotype data and genotype data filtered after Plink 1.9 were read into Tassel 2.1 for correlation analysis. A generalized linear model (GLM) and mixed linear model (MLM) were applied to assess the optimal model^34^. Manhattan and quantile–quantile (Q–Q) plots of the GWAS data for six indices were generated using R 3.12 and the R package ‘qqman’^36^. By comparing the Q-Q plots and Manhattan maps generated by the two models, it was found that the MLM was the most suitable model, which outperformed the GLM model in terms of association accuracy and control for false positives and false negatives. The recommended threshold was set at 1.41 × E-06 (0.05/35,430)^37^. Genetic loci and gene information were sourced from the B73 reference genome RefGen_V4 (http://www.maizegdb.org/). Homologous gene annotations were retrieved from NCBI (http://www.ncbi.nlm.nih.gov/), RAP-DB (http://rapdb.dna.affrc.go.jp/), and TAIR (https://www.arabidopsis.org/) databases.

### Gene expression analysis in different inbred lines

We used three inbred sweet corn lines, namely 307#, 19hi135, and 20hi111, and germinated them at 10 ℃. Maize seed samples were collected at 0, 1, 3, 5, 7, and 10 days. Ten seeds were one replicate, and a total of three replicates were taken. Total RNA was extracted using the quick RNA isolation kit (Huayueyang Biotechnology Co., Ltd., Beijing, China) per manufacturer’s instructions. The first strand of cDNA was synthesized using TransScript Uni All-in-One First-Strand cDNA Synthesis SuperMix (TransGen Biotech, Beijing, China). Primer3 Plus software was used to design qRT‒PCR primers (Table S2). *ZmActin* served as the internal control. For qRT-PCR detection, TB Green Pre mixed Ex Taq II (Takara Bio, Beijing, China) and the QuantStudio 5 real-time PCR instrument (Thermo Fisher Scientific, Waltham, MA, USA) were employed. Data analysis was conducted using the quantification method (2^−ΔΔCT^) and three replicates were used to calculate the relative expression level^38^.

### Statistical analysis

Data processing and mapping were undertaken using Microsoft Excel 2023 (Microsoft, Redmond, Washington D.C., USA) and Origin 2021 (Origin Lab, Northampton, MA, USA). One-way analysis of variance (ANOVA) and mean values were performed on the data using SPSS 14.0 (International Business Machine, Chicago, IL), followed by Duncan’s multiple range test and the least significant difference (LSD) test. Significant differences were identified at the *P* < 0.05 threshold.

## Results

### Phenotypic evaluation

**Figure 1:**
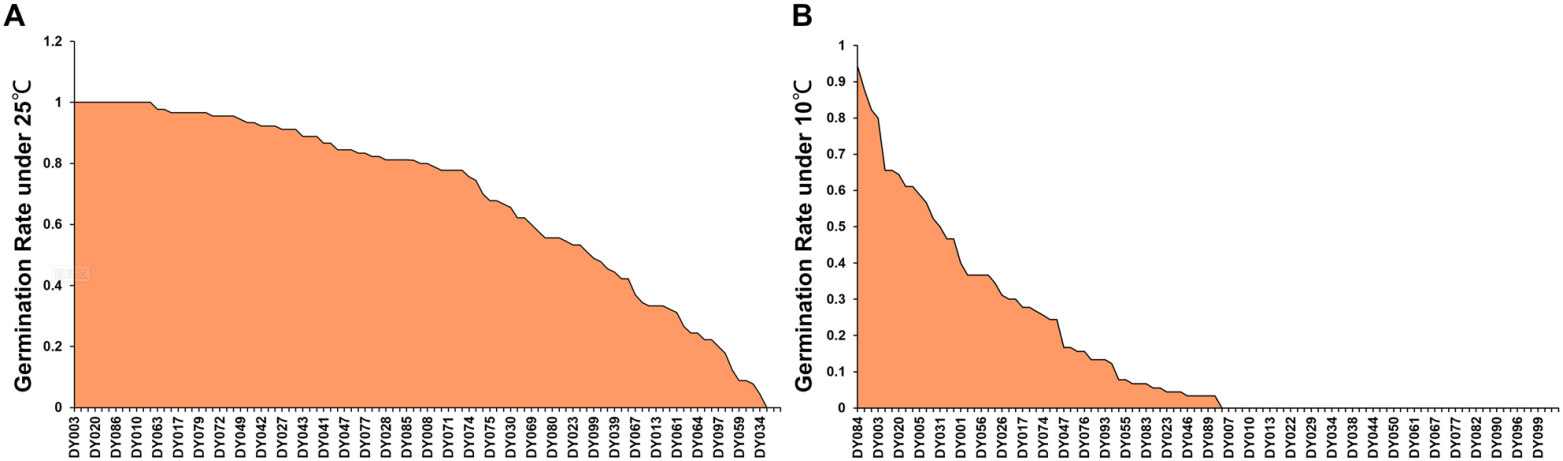
Seed germination rate of 100 sweet corn inbred lines under 25 ℃ (**A**) and 10 ℃ (**B**). The germination rates of 100 sweet corn inbred lines are sorted from high (left) to low (right), and several names of inbred lines are displayed on the horizontal axis.

The germination rate of diverse sweet corn germplasm varied under room temperature (25 ℃) (Table 1, Fig. 1A). The germination rate at room temperature (R) ranged from 0.04 to 1.00, with a median of 0.8 and an average of 0.7. The germination rate of 12 inbred lines all reached 1.00, including 19hi135 (DY084), 19hi534 (DY003), and 20hi169 (DY010) and the inbred line1079Z/SU (DY034) had the lowest germination rate of 0.04 (Fig. 1A, Table S3). The ANOVA table can be seen in Table S4. The germination indicators showed significant differences under different temperature conditions, genotypes, and the interaction between genotypes and temperature conditions, and there was no significant difference between each repetition (Table S4). Extreme variations in germination rates under chilling treatment (GR) were observed for inbred lines (Table 1, Fig. 1B). The range of GR was between 0.00 and 0.94, with the average directly decreasing to 0.22 and the median decreasing to 0.07. 19hi135 (DY084) exhibited the highest germination rate at 94.44%. The germination rate for the three inbred lines M688 (DY002), CHLYF (DY014), and 19hi534 (DY003)) surpassed 80%. However, 37 varieties failed to germinate within 21 days, and no germination was observed even with extended observations (Fig. 1B, Table S3). The germination index under 25 ℃ (I) ranged from 0.75 to 32.07, averaging 17.34 (Table 1). However, the germination index under 10 ℃ (GI) ranged from 0 to 18.47, with an average of 2.24 and the RGI ranged from 0 to 0.84, averaging 0.10.

**Table 1:** Descriptive statistics for germination-related traits at 25 and 10 °C.

Trait	N	Mean	Median	SD	Min	Max
R	100	0.70	0.80	0.28	0.04	1.00
GR	100	0.18	0.07	0.24	0.00	0.94
RGR	100	0.22	0.12	0.26	0.00	0.94
I	100	17.34	18.43	8.10	0.75	32.07
GI	100	2.24	0.45	3.71	0.00	18.47
RGI	100	0.10	0.03	0.15	0.00	0.84
T	100	6.18	5.97	1.64	3.19	9.89
DT	100	23.26	24.42	20.41	0.00	67.99
RDT	100	4.28	4.33	4.02	0.00	14.49

Descriptive statistical data includes sample size (N), mean, median, standard deviation (SD), minimum value (Min), and maximum value (Max). One hundred sweet corn inbred lines were used for germination experiments at room temperature (25 ℃) and low temperature (10 ℃). The experiment was repeated three times and the average value was taken. Directly observed traits included germination rate on the 7th day under room temperature (R), germination index on the 7th day under room temperature (I), average germination time on the 7th day under room temperature (T), germination rate on the 21st day under low temperature (GR), germination index on the 21st day under low temperature (GI), average germination time on the 21st day under low temperature (DT), relative germination rate (RGR), relative germination index (RGI), and average germination time (RDT).

The average germination time at 25 ℃ (T) spanned 3.19–9.89, averaging 6.18. The average germination time under 10 ℃ (DT) significantly extended, which ranged from 0.00 to 67.99, averaging 23.26. And the relative average germination time (RDT) ranged from 0.00 to 14.49.

**Figure 2:**
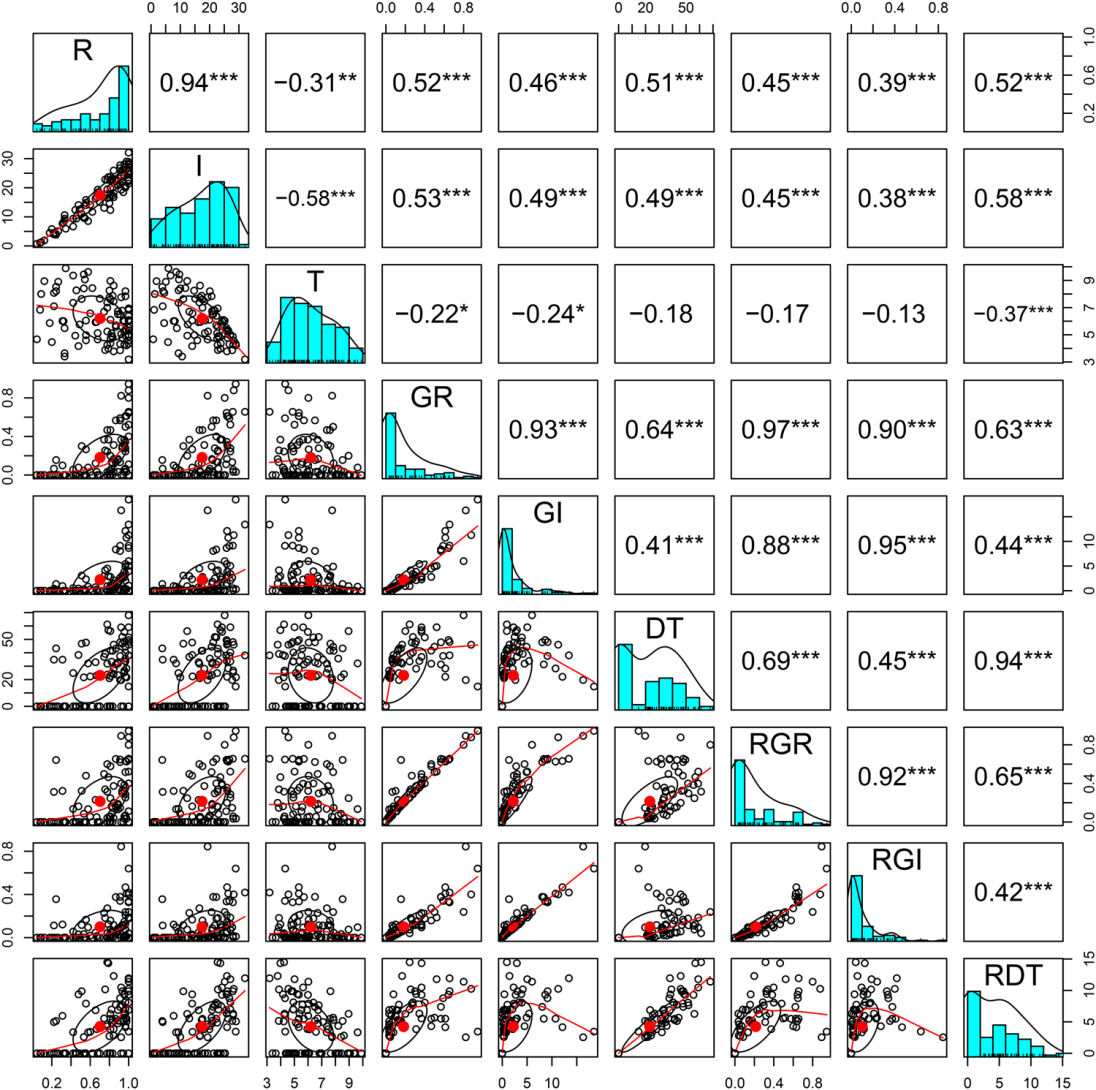
Distribution and correlation of nine indicators related to chilling-tolerant germination of sweet corn seeds. The histogram on the central diagonal represents the frequency distribution of each indicator, the numbers in the inverted triangle block represent the Spearman coefficients between each pair of indicators, the scatter plot in the regular triangle block represents the exponential correlation and distribution pattern, and the red line represents the correlation trend.

In total, we assessed nine traits, including six traits and three relative traits under two treatments (25 and 10 ℃), and the distribution patterns and correlations of these traits are depicted in Fig. 2. Except for DT and RDT, which displayed weak bimodal distributions, all other traits exhibited unimodal distributions. The Spearman correlation coefficient indicated a significant correlation between germination rate and germination index at both room and low temperatures. For instance, the correlation coefficients between R and I were 0.94, between GR and GI were 0.93, and between RGR and RGI was 0.92. The correlations between indicators under cold conditions and their relative indicators were robust, while those between indicators under cold and normal temperature conditions were weak. For example, the correlation coefficient between GR and RGR was 0.97, whereas that between T and DT was only − 0.18.

### Principal component analysis, relative kinship, LD decay, and phylogenetic analysis

**Figure 3:**
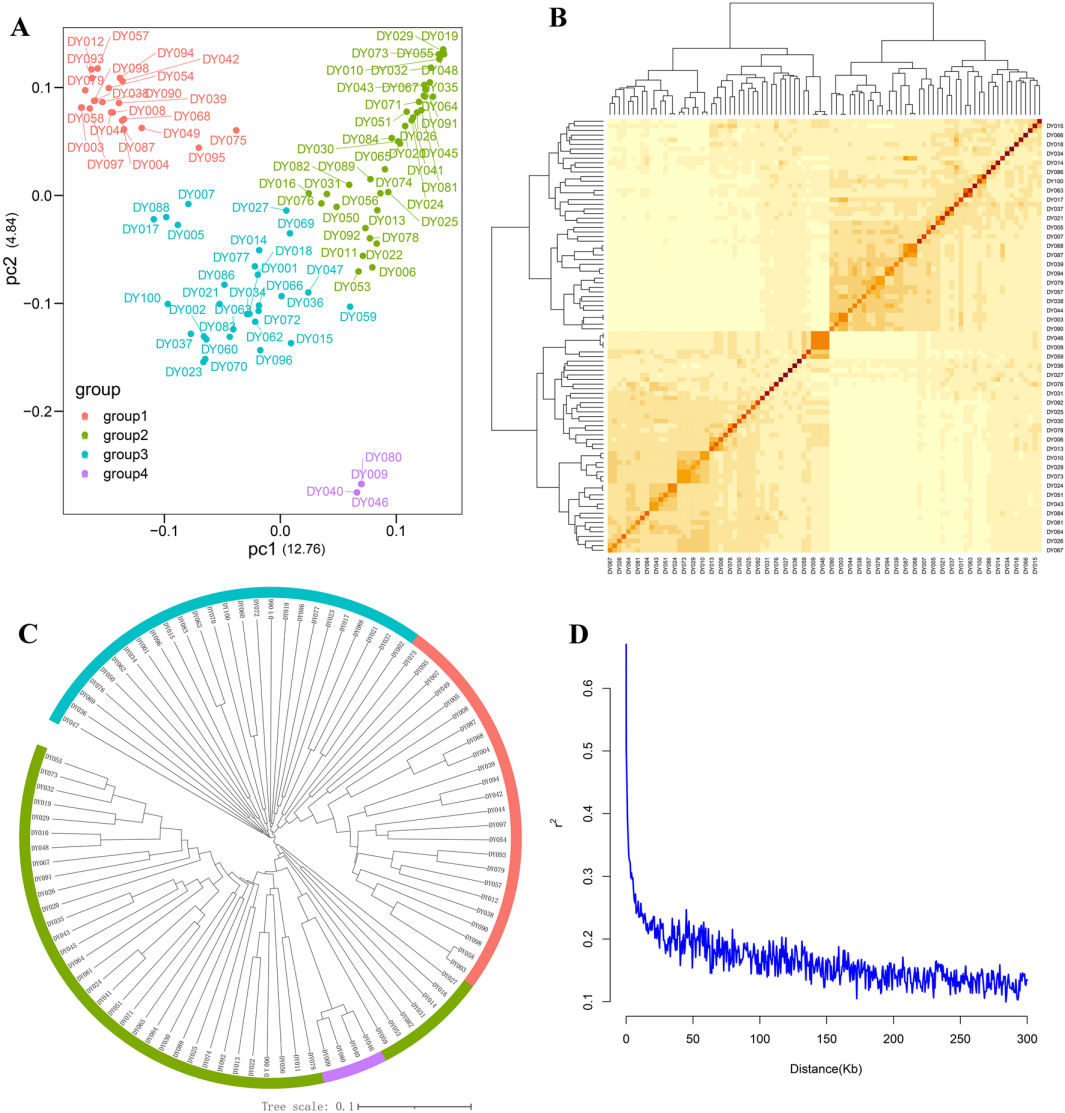
Principal component analysis (PCA), relative kinship, linkage disequilibrium (LD) decay, and phylogenetic analysis of sweet corn inbred lines in the association panel. (**A**) PCA. (**B**) Relative kinship heatmap of the sweet corn accessions. (**C**) Phylogenetic tree of sweet corn associations based on SNPs. (**D**) LD of the sweet corn accessions.

To comprehend the population structure of sweet corn germplasms, a PCA chart and a genetic relationship matrix were constructed. Ninety-four sweet corn germplasm lines were categorized into four subgroups (Fig. 3A). Based on known lineage information and geographical origins, the first subgroup comprised mostly tropical inbred lines (22 inbred lines), the second subgroup comprised 29 temperate inbred lines, the third subgroup comprised 39 inbred lines of temperate hybrid (subtropical) origin, and the fourth subgroup represented American line material, comprising four inbred lines selected from American hybrids. The correlation between different germplasms was weak and did not impact subsequent correlation analysis (Fig. 3B). A phylogenetic tree constructed using the maximum-likelihood method again categorized the 94 lines into four groups, consistent with the PCA results (Fig. 3C). The LD results of the 35,430 SNP analysis groups showed that when the *r*^2^ cutoff value was set to 0.2, the average LD attenuation distance of the chromosomes was 50 kb (Fig. 3D).

### Genetic loci identification and functional classification of genes regulating chilling-tolerant germination in sweet corn

**Figure 4:**
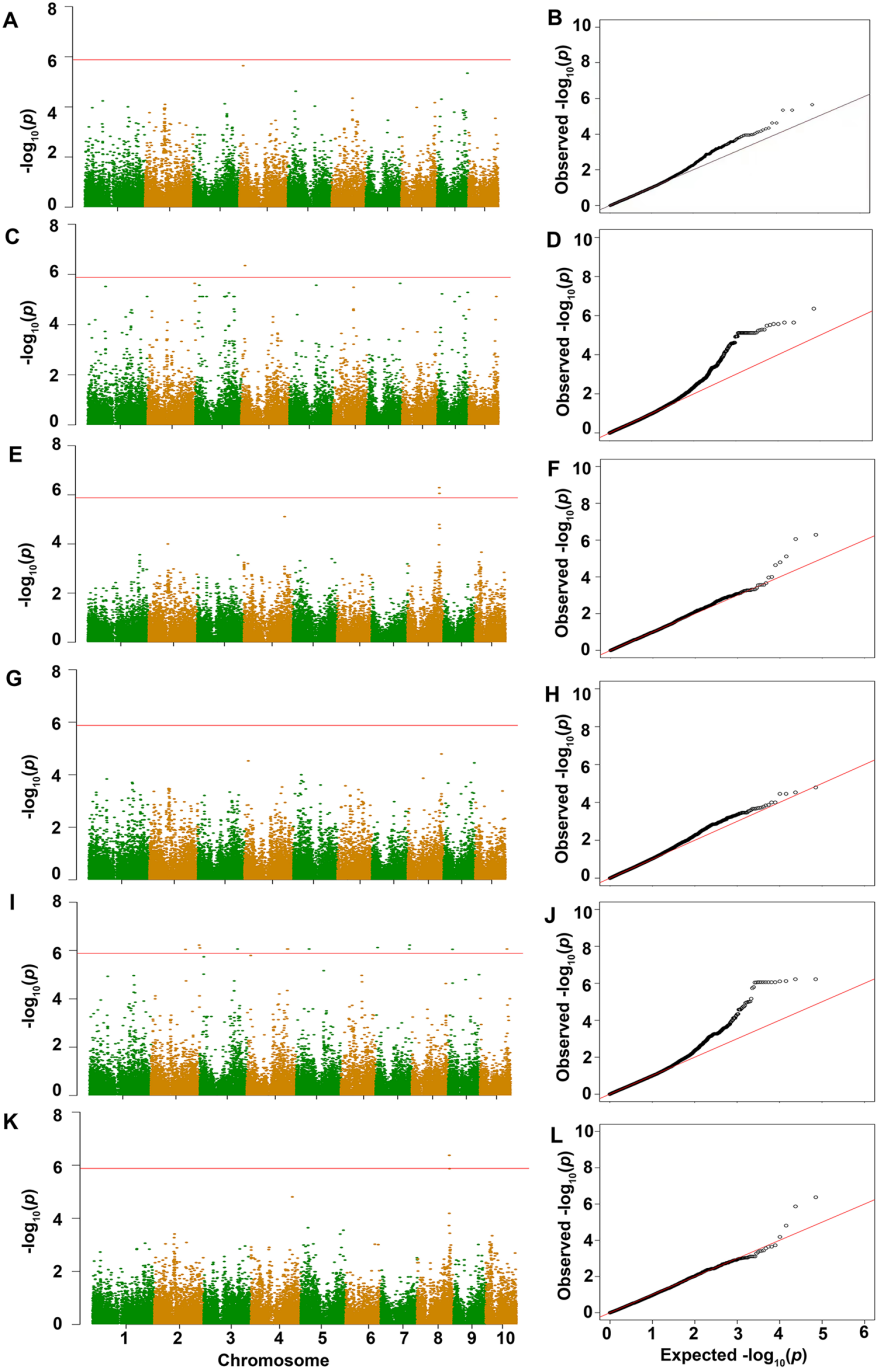
Manhattan (left) and quantile–quantile (Q–Q) plots (right) of the GWAS results for six indices related to chilling-tolerant germination in sweet corn. (**A**,**C**,**E**,**G**,**I**,**K**) represent the association signals in the entire genome of all 10 chromosomes of GR, GI, DT, RGR, GRI, and RDT from top to bottom. The horizontal axis represents the chromosome position, and the vertical axis represents the observed − log10P. The red line represents the threshold. (**B**,**D**,**F**,**H**,**J**,**L**) represent the correlation between the observed signals (black dots) and the expected signals (red dots) of GR, GI, DT, RGR, GRI, and RDT from top to bottom, with the horizontal axis representing the expected value and the vertical axis representing the observed value.

Six indicators that are significantly related to seed cold tolerance germination (GR, GI, DT, RGR, RGI, and GDT) were subjected to GWAS analysis, with the Bonferroni threshold located at 1.41 × E-06 (− log10 *P* = 5.85). We shortlisted 16 SNPs that surpassed the threshold, all exhibiting a MAF exceeding 0.1 (Fig. 4, Table 2). The most strongly correlated SNP was AX-86297104, with a *P*-value of 5.11E-07, correlating with both DT and RDT, and AX-86302170 followed closely.

**Table 2:** Significant SNPs associated with germination.

SNP	Chromosome	Position	MAF	*P*-value in					
				GR	GI	DT	RGR	RGI	RDT
AX-86297104	8	158788019	0.3292			5.11E-07			4.23E-07
AX-86302170	4	18469666	0.1504		4.42E-07				
AX-86261150	2	240323548	0.1257					5.98E-07	
AX-116876284	7	170105222	0.0277					5.98E-07	
AX-86317698	7	9653517	0.1					7.55E-07	
AX-86238955	2	244411542	0.0627					7.83E-07	
AX-86311867	3	188251800	0.0817					8.67E-07	
AX-86313987	4	201401844	0.0554					8.67E-07	
AX-86328878	4	202671725	0.018					8.67E-07	
AX-86291616	4	204466334	0.0207					8.67E-07	
AX-86271769	5	63460329	0.1551					8.67E-07	
AX-86271780	5	64079062	0.1281					8.67E-07	
AX-86274990	7	167712767	0.1174					8.67E-07	
AX-86299373	10	134110205	0.018					8.67E-07	
AX-86304707	9	21411460	0.0042					8.94E-07	
AX-86309806	2	172074580	0.0597					9.03E-07	

The B73 RefGen v4 maize gene database^27^ (http://www.maizegdb.org/) facilitated the identification of candidate genes directly affected by relevant SNPs or those exhibiting high LD in proximity. By screening genes within a 50 kb distance of LD (*r*^2^ ≥ 0.2) from 16 related SNPs, we identified 46 candidate genes (Table S1).

Six candidate genes crucial for cold germination tolerance was chosen, and orthologous genes in Arabidopsis and rice were identified (Table 3). *Zm00001d052854* encodes a pentatricopeptide repeat-containing protein mitochondrial. *Zm00001d052855* encodes a heat shock protein 13 (HSP13) protein. *Zm00001d049161* encodes 11-beta-hydroxysteroid dehydrogenase-like 5. *Zm00001d011687* encodes IAA-amino acid hydrolase ILR1-like 4. The homologous genes of the four genes were related to seed germination in Arabidopsis. *Zm00001d014794* (physical impedance-induced protein 2) and *Zm00001d045417* (2-oxoglutarate (2OG) and Fe (II)-dependent oxygenase superfamily protein) were linked to low-temperature stress response.

**Table 3:** Six candidate genes associated with germination under cold condition.

SNP	Gene	Annotation	Abbreviation	*Arabidopsis thaliana*	*Oryza sativa*	Function
AX-86328878	Zm00001d052854	Pentatricopeptide repeat-containing protein mitochondrial	ZmPPR	AT2G44880	Os08t0500600	Seed swelling and development
AX-86328878	Zm00001d052855	Heat shock protein13	ZmHSP13	AT5G56030	Os08t0500700	Heat acclimation, leaf development, negative regulation of seed germination
AX-86302170	Zm00001d049161	11-beta-hydroxysteroid dehydrogenase-like 5	Zm11βHSDL5	AT5G50700	Os11t0499600	Proteome of oil bodies from mature seeds
AX-86271769	Zm00001d014794	Physical impedance induced protein2	ZmPIIP2	AT2G17840	Os06t0717100	Upregulation under strong light, drought, cold, and salt stress
AX-86304707	Zm00001d045417	2-oxoglutarate (2OG) and Fe(II)-dependent oxygenase superfamily protein	Zm2OGO	AT4G36090	Os06t0138200	Responsive to stress conditions
AX-86297104	Zm00001d011687	IAA-amino acid hydrolase ILR1-like 4	ZmILL4	AT1G51760	OS01T0706900	Regulating the homeostasis of auxin and jasmonic acid

### Candidate gene expression pattern in different inbred lines during low-temperature germination

**Figure 5:**
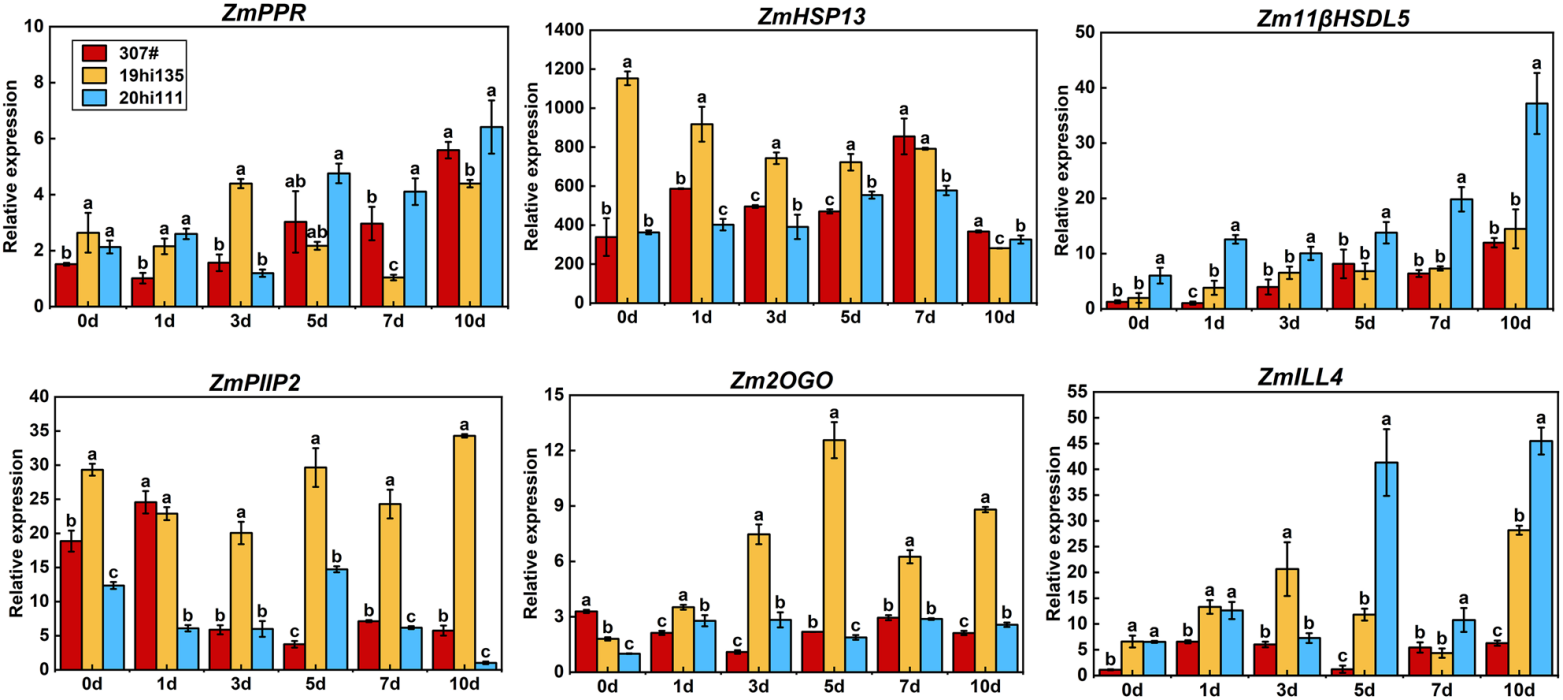
Expression patterns of 6 candidate genes during low-temperature germination in the cold-tolerant inbred line 19hi135 (R = 1.00, GR = 0.944), moderately cold-tolerant inbred line 307# (R = 0.911, GR = 0.589), and cold-sensitive inbred line 20hi111 (R = 0.922, GR = 0). Standard error is indicated. Different letters represent significant differences as determined using one-way ANOVA followed by Duncan’s test. *P* < 0.05.

To validate the role of the candidate genes in sweet corn germination at low-temperature, we selected the cold-tolerant inbred line 19hi135 (R = 1.00, GR = 0.944), moderately cold-tolerant inbred line 307# (R = 0.911, GR = 0.589), and cold-sensitive inbred line 20hi111 (R = 0.922, GR = 0) as experimental material. Samples were collected after 0, 1, 3, 5, 7, and 10 days of germination at low temperature (10 ℃) to assess the expression patterns of candidate genes. Results of qRT-PCR revealed a continuous increase in *Zm11βHSDL5* expression at 10 ℃; the gene expression was consistently significantly higher in 20hi111 than in 307# and 19hi135. The expression of *Zm2OGO* initially increased and then decreased in 19hi135, but remained significantly higher than that in 307# and 20hi111(Fig. 5). This indicated that *Zm11βHSDL5* and *Zm2OGO* likely play negative and positive regulatory roles in the low-temperature germination process of sweet corn, respectively.

## Discussion

Low temperature is a primary abiotic stressor impacting maize seed germination, reproduction, and biomass accumulation^39^. Because of weather fluctuations, chilling threatens sweet corn production. Sweet corn can be used as both food and vegetables and is widely popular in the consumer market. Farmers sow it in early spring to increase economic benefits. However, this will require higher seed vitality, faster germination time, and better germination rates under chilling conditions. This makes chilling-tolerance breeding of maize an important strategy, and it has been feasible to improve maize chilling-tolerance by modifying related genes. Therefore, it is very important to explore genes related to cold tolerance.

GWAS has become an important method for locating key candidate genes^40^. Chilling tolerance is a complex quantitative trait. Several GWAS have explored the molecular mechanisms underlying chilling tolerance in maize. Under chilling stress, SNPs linked to the RGR were identified on chromosomes 1, 2, 4, and 7, with a specific SNP related to the relative number of roots during germination located on chromosome 2^41^. A joint analysis employing GWAS and RNA-seq uncovered two candidate genes associated with mitogen-activated protein kinase (MAPK) signal transduction and fatty acid metabolism, both integral to cold germination tolerance^42^. Here, we analyzed GWAS data using 94 sweet corn inbred lines to identify genes linked to chilling-tolerant germination. Utilizing a 56K gene chip for SNP detection, capable of identifying 56,000 SNP loci with multiple effective markers, high quality, and stability, we harnessed the potential of this third-generation molecular marker in various aspects of crop breeding, including genetic map construction, germplasm resource analysis, biodiversity detection, and linkage imbalance association analysis^30,43^. For instance, Hu et al. recently amalgamated and refined a GBS dataset of SNP markers from an Illumina corn 50K array and SNP markers retrieved from the database^3^. It makes genotyping higher quality, and more efficient and stable.

The germination rate of different sweet corn inbred lines varies greatly at room temperature (Fig. 1A). Mutations in genes regulating starch synthesis and related metabolism pathways impede starch synthesis in sweet corn, leading to sugar (mainly sucrose) accumulation. Starch deficiency can to some extent affect the germination of sweet corn seeds^44^. Sweet corn is not as abundant as ordinary corn in terms of germplasm resources. The 100 inbred lines with different genetic backgrounds used in this study were accumulated by our team members through more than 20 years of breeding work. We observed that the germination time of corn seeds increases as the temperature decreases. It has been reported that 10 ℃ marks the lower limit for corn seed germination^45^. Therefore, we set 10 °C as the experimental temperature for cold resistance treatment. During the low-temperature treatment, only 14 varieties attained a germination rate exceeding 50% by day 21; notably, 37 varieties failed to germinate at 10 ℃ (Fig. 1). This was much lower than the germination rate of ordinary corn at low temperatures^3,46^. 19hi135 (DY084) and M688 (DY002) exhibited high germination rate under normal and low temperature. Conversely, 20hi169 (DY010), 20hi127 (DY011), and JBT15 (DY012) displayed a high germination rate of 1.00 under standard conditions but failed to germinate at 10 ℃, revealing their high susceptibility to cold stress. Excellent chilling-tolerant inbred lines, such as 19hi135, can be used as ideal germplasm resources for mechanism research and germplasm improvement.

In this study, we observed robust correlations among individual germination traits (Fig. 2). Notably, the correlation between germination traits at room temperature and low temperature was weak, suggesting that distinct genetic factors may govern germination under different temperatures. However, a strong correlation (> 90%) was evident for germination rate and germination potential at room and low temperatures, as well as their relative traits (RGR and RGI). This finding aligns with previous results reported in 2017^3^. Based on the results of association analysis, we identified 16 significant SNPs associated with germination (Table 2) and shortlisted 46 candidate genes (Table S1). Subsequent comparisons with homologous proteins in Arabidopsis and rice unveiled six candidate genes associated with germination in cold conditions (Table 3). We found that some strongly correlated phenotypic traits did not correspond to common SNP locus. We attempted to lower the Bonferroni threshold to 1 × E-05 to screen for more SNP sites. The results showed that nine SNPs were significantly correlated with multiple indicators. Four SNPs were significantly associated with GI and RGI, showing strong correlations. Two indicators were significantly correlated with DT and RDT. Two indicators were significantly correlated with GR and GI. An SNP (AX-86302170) was significantly associated with both GR, GI, and RGI (Table S5). We found that there may be differences in the *P*-value range after association analysis for each trait, but because of the uniform threshold, the number of SNPs screened for each trait may vary, and there may not even be loci higher than the threshold. Once the threshold is lowered, the common SNP loci between strongly correlated traits will increase.

The AX-86302170 locus, associated with GI, harbors the gene *Zm00001d049161 (Zm11βHSDL5)*, encoding 11-beta hydroxysteroid dehydrogenase-like 5. The homologous gene in Arabidopsis and rice, *HSD1*, shows specific and highly induced transcription in oil-accumulating tissues of mature seeds^47^. Transgenic Arabidopsis plants overexpressing *AtHSD1* exhibit reduced seed dormancy, decreased sensitivity to abscisic acid (ABA), and increased decomposition^48^. *OsHSD1* expression is induced by cold stress^49^, and its homologous gene in *Brassica napus* showed higher expression levels in non-germinated seeds compared to germinated seeds following treatment with ABA analogue PBI429^50^. In this study, *Zm11βHSDL5* expression increased with prolonged incubation at low temperature and was higher in the cold-tolerant inbred line than in the cold-sensitive inbred line. These results align with the performance of *OsHSD1* at low temperatures. Based on the expression pattern, we speculate that *Zm11βHSDL5* may negatively regulate sweet corn germination at low temperatures. However, given *AtHSD1’s* positive role in Arabidopsis seed germination, further research is warranted. *Zm00001d045417* encodes 2OG and Fe (II)-dependent oxygenase superfamily proteins, and its homolog in Arabidopsis is activated during seed germination, responding to cold stress and ABA treatment^51^. *Zm00001d052854* encodes a mitochondrial pentatricopeptide repeat-containing protein, and its Arabidopsis homolog ABA hypersensitive genesis 11 is differentially expressed during seed swelling. Importantly, its germination is ABA-sensitive^52,53^. Z*m00001d014794* expression is associated with cold stress^54^. The *Zm00001d052855* homolog in Arabidopsis encodes heat shock protein HSP90.2 and negatively regulates seed germination^55^. *Zm00001d011687*, characterized by the SNP site AX-86297104, participates in auxin metabolism^56^. Overall, these candidate genes need further evaluations to elucidate their roles in chilling-tolerant germination of sweet corn.

## Conclusions

The data obtained in this study facilitated population structure analysis and the establishment of a novel sweet corn panel, accompanied by a phylogenetic tree. Six germination indicators (GR, GI, DT, RGR, GRI, and GDT) were employed for GWAS analysis, resulting in the identification of 16 significantly correlated SNPs. Notably, nine single SNPs exhibited significant associations with multiple germination traits. Within 50 kb upstream and downstream of these SNPs, 46 related candidate genes associated with germination were identified, and eventually six candidate genes related to cold germination tolerance in sweet corn were shortlisted. The expression patterns of these candidate genes indicated that *Zm11βHSDL5* and *Zm2OGO* likely exert a negative and positive regulatory influence, respectively, on the low-temperature germination of sweet corn. These discoveries furnish crucial theoretical underpinnings for comprehending the biological mechanisms governing low-temperature germination in sweet corn and expediting advancements in breeding practices. Furthermore, this study establishes a groundwork for the creation of specialized germplasm resources tailored for low-temperature-tolerant germination in sweet corn.

### Supplementary Information

Supplementary Table S1.

Supplementary Table S2.

Supplementary Table S3.

Supplementary Table S4.

Supplementary Table S5.

## Data Availability

The data reported in this paper have been deposited in the China National Center for Bioinformation/Beijing Institute of Genomics, Chinese Academy of Sciences (https://ngdc.cncb.ac.cn/?lang=en, accession no. PRJCA022770).

## Abbreviations

CTAB: Cetyltrimethylammonium bromide
LOD: Logarithm of odds
MAF: Minor allele frequency
MAPK: Mitogen-activated protein kinase
PCA: Principal component analysis
QTLs: Quantitative trait loci
SNP: Single-nucleotide polymorphism
GWAS: Whole-genome association study
